# Nuclear disaster and the medical problems during the earthquake in Japan, 2011

**DOI:** 10.1186/cc11093

**Published:** 2012-03-20

**Authors:** Y Haraguchi, Y Tomyasu, H Nishi, M Sakai, E Hoshino, M Hoshino, T Takeda-Nozawa

**Affiliations:** 1 Japanese Compendium Team for Disaster Medicine, Kawasaki, Japan

## Introduction

The roles of medicine including intensivists against natural mega-disaster followed by artificial disaster are discussed.

## Methods

The Higashinihon earthquake caused more than 2,000 deaths or missing, which was followed by the Fukushima Daiichi nuclear plant explosion. This study was mainly studied based upon on the actual experience in and around the nuclear station.

## Results

Many medical teams, rescue teams and public officials worked hard. However, many serious problems are revealed, even if they are limited to the medical fields, which are as follows: inappropriate basic preparedness against the largest degree of mega-disaster; lack of official education for medical teams against special disaster, such as nuclear disaster (that is, most members of the Japan DMAT or disaster medical assistance team seemed to be laypersons); incorrect standard/rules of Japan DMAT, which were thought to be excessively focused upon the cure of the injured patients and a planned short period or nearly 48 hours; and insufficient consideration for the weak people or CWAP: children, (pregnant) women, aged people, and the poor people/sick patients. Many CWAP seemed not to have survived.

## Conclusion

In order to cope with the mega-disaster, it became evident that it is insufficient to take makeshift measures or use cheap tricks. Working out the systematization of disaster medicine, based upon the academic viewpoints and philosophy/reliability, is essential to protect the people and the nation too.

